# Correction: Design, synthesis, and evaluation of bitopic arylpiperazine-phthalimides as selective dopamine D_3_ receptor agonists

**DOI:** 10.1039/c8md90042f

**Published:** 2018-09-11

**Authors:** Yongkai Cao, Ningning Sun, Jiumei Zhang, Zhiguo Liu, Yi-zhe Tang, Zhengzhi Wu, Kyeong-Man Kim, Seung Hoon Cheon

**Affiliations:** a Integrated Chinese and Western Medicine Postdoctoral Research Station , Jinan University , Guangzhou 510632 , China; b College of Pharmacy and Research Institute of Drug Development , Chonnam National University , Gwangju 500-757 , Republic of Korea . Email: kmkim@jnu.ac.kr ; Email: shcheon@jnu.ac.kr ; Fax: +82 625302949 ; Fax: +82 625302911 ; Tel: +82 625302936 ; Tel: +82 625302929; c The Fist Affiliated Hospital of Shenzhen University , Shenzhen 518035 , China; d Chemical Biology Research at School of Pharmaceutical sciences , Wenzhou Medical University , Wenzhou 325035 , China . Email: szwzz001@163.com ; Fax: +86 75525622938 ; Tel: +86 75525622938

## Abstract

Correction for 'Design, synthesis, and evaluation of bitopic arylpiperazine-phthalimides as selective dopamine D_3_ receptor agonists' by Yongkai Cao *et al.*, *Med. Chem. Commun.*, 2018, DOI: 10.1039/c8md00237a.



## 

The authors regret the following errors in their manuscript: in the Abstract, in the sentence beginning ‘Molecular docking demonstrated…’, ‘Phe^7.39^’ should be changed to ‘Tyr^7.35^’. In section ‘2.4 Molecular basis of selectivity over D2R’, in the sentence beginning ‘Notably, the different orientations…’, ‘Phe365/429’ should be changed to ‘Tyr365/429’. In section ‘3. Conclusion’, in the sentence beginning ‘The different orientations…’, ‘Phe^7.39^’ should be changed to ‘Tyr^7.35^’.

The Royal Society of Chemistry apologises for these errors and any consequent inconvenience to authors and readers.

